# Comparing Older Parents’ and Adult Children’s Fear of Falling and Perceptions of Age-Friendly Home Modification: An Integration of the Theories of Planned Behavior and Protection Motivation

**DOI:** 10.3390/bs13050403

**Published:** 2023-05-11

**Authors:** Hyun Joo Kwon, Jiyoung Oh

**Affiliations:** 1 Department of Interior & Environmental Design, Pusan National University, Busan 46241, Republic of Korea; hyunjookwon@pusan.ac.kr; 2 Research Institute of Ecology, Pusan National University, Busan 46241, Republic of Korea

**Keywords:** fear of falling, home modification, older adults, adult children, theory of planned behavior, protection motivation theory

## Abstract

This study investigated how a fear of falling affects perceptions and behavioral intentions toward age-friendly home modification (AFHM) in older parents and adult children by integrating the theory of planned behavior (TPB) to explain AFHM decision-making processes and the protection motivation theory to explain the impact of a fear of falling on AFHM intention. The target population comprised older parents (≥75 years old) and adult children (45–64 years old) in Busan, South Korea (N = 600). The participants completed a self-administered questionnaire in March 2022. Independent *t*-test and path model analyses were conducted to compare primary constructs between older parents and adult children and analyze the relationships among a fear of falling, TPB components, and AFHM intention. Results showed that both groups had positive attitudes toward AFHM. However, adult children showed significantly higher rates of having a fear of falling, lower perceived behavioral control, and higher AFHM intention than older parents. The proposed research models were partially supported in the older-parent group and fully supported in the adult-children group. Adult children play a critical role in AFHM, along with older adults who are directly involved in an aging society. AFHM-supporting programs, including monetary and human-force assistance, education, related public advertisements, and an active AFHM market, should be expanded.

## 1. Introduction

People are more likely to experience falls as they grow older [1]. Given their physical weaknesses, falling can lead to prolonged recovery or cause death in older adults [2]. Senior falls degrade their quality of life and increase medical expenses, which add to the burden of social costs [3]. The main reasons for senior falls are physical decline due to aging and built environments that cannot support such health changes [4]. Most senior falls occur at home. Every year in the United States, 30% of individuals aged ≥65 years and 50% of individuals aged ≥80 years experience falls [5]. In South Korea, the fastest-aging country in the world [6], 60.5% of people aged ≥65 years fall in their homes [7]. An unsafe living environment increases the risk of falls and reduces physical activity levels as a result of an increased fear of falls, which directly restricts successful aging in place (i.e., remaining in the current housing for as long as possible) [8,9]. Older people who have experienced falls have a greater fear of falls [10], resulting in mobility restrictions and depression, which negatively affect their physical and psychological health [11,12,13].

Although older adults experience physical aging, they often continue living in houses where they have lived since their youth, which lack age-friendly design features [14]. As the aging of the population becomes a global phenomenon, world-leading organizations, such as the World Health Organization [15] and the United Nations [16], have exerted efforts toward creating “age-friendly” environments to improve the quality of life for older adults. The design of age-friendly housing, an essential environment for human beings, is crucial for a safe and healthy later life. Older adults know that their current houses need to be renovated [17,18] to support the physical constraints of aging, which can be defined as age-friendly home modification (AFHM). In particular, people aged ≥80 years living in poor housing conditions are likelier to need age-friendly housing and modify their homes [19]. However, there are various barriers for older adults, such as costs and a lack of knowledge [17,20] or information on the type of home modification that supports aging [19,21]. If the government had a proper home modification policy for older adults, they could access these services. However, in cases where there is no government support program or such a program requires a patient to meet beneficiary criteria, it might be difficult for older adults to renovate their housing without the help of a third party.

Family members, including adult children, are among the closest people to contact older adults regarding home modification [22]. Although fewer people live with their older parents [2,23], adult children’s opinions greatly influence significant decisions related to the daily lives of older adults [24,25]. Previous studies have shown that adult children’s opinions significantly influence their parents’ decisions about caregiver choice [26] and wheelchair purchase [24]. In housing-related choices for older adults in later life, older parents ask their children for help, and their children play an essential role in choosing home modifications [8]. Therefore, the decision-making processes related to older adults’ home modification must be understood among not only older adults but also their children.

Ajzen’s theory of planned behavior (TPB) is a representative theory that explains the process of determining specific behaviors and has been applied to explain individual intentions for various health-related behaviors [27,28]. It has also been used as an extended theory to understand the decision-making processes of older adults regarding aging in place [29] or to predict the home-replacement behavioral intentions of older adults moving to an assisted living facility [30].

Rogers and Prentice-Dunn’s protection motivation theory (PMT) explains the process by which a fear of falling leads to safety behaviors [31]. It explicates how threats and coping appraisals cause behavioral change. The PMT is used to understand older adults’ fear of falling and their fall-protection behaviors [32] and to examine the psychosocial correlation between this and parents’ safety behaviors regarding toddler falls [33].

Some studies have been conducted on the perceptions and intentions of older adults toward in-home modification in later life [18,19]. Studies on adult children’s views on their parents’ housing environments in later life are limited. For example, Marquardt et al. conducted a study on the role of adult children as family caregivers in their parents’ home modification [22]. However, there are very few studies on the perceptions and intentions of adult children who do not live with their parents toward the parents’ home modification. In particular, there are insufficient studies on home modification for older parents that examine differences in perceptions between older parents and adult children and identify the two groups’ decision-making processes regarding such modifications. The current study addresses this gap and seeks ways to implement home modification for older adults effectively.

### 1.1. Purpose of the Study

This study aims to investigate how a fear of falling affects perceptions and behavioral intentions toward AFHM in older parents and adult children by integrating the TPB and PMT. We compared the fear of falling, three TPB components (i.e., attitude, subject norm, and perceived behavioral control toward AFHM), and AFHM intention between the two groups. We also analyzed relationships among the fear of falling, the three aforementioned TPB components, and AFHM intentions in both groups. We focused on older parents’ perceptions toward their own fear of falling and AFHM and those of adult children toward their parents.

### 1.2. Hypotheses

**Figure 1:**
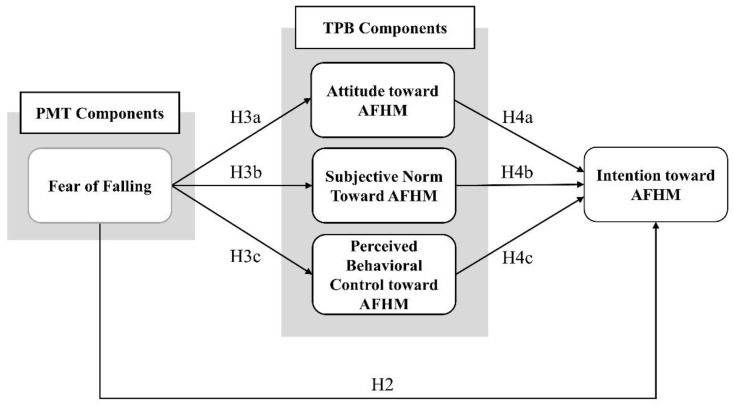
Conceptual model for intention toward age-friendly home modification.

Based on the conceptual model (Figure 1), we developed the following hypotheses:

Hypotheses Regarding the Comparison Between Older Parents and Adult Children

**H1.**  *There are significant differences between older parents and adult children regarding a fear of falling, TPB components, and AFHM intention.*

**H1a.**  *There is a significant difference between older parents and adult children regarding a fear of falling.*

**H1b.**  *There is a significant difference between older parents and adult children regarding their attitudes toward AFHM.*

**H1c.**  *There is a significant difference between older parents and adult children regarding their subjective norms toward AFHM.*

**H1d.**  *There is a significant difference between older parents and adult children regarding their perceived behavioral control toward AFHM.*

Hypotheses Regarding the Proposed Research Model

**H2.**  *A fear of falling significantly affects AFHM intention.*

**H3.**  *A fear of falling significantly affects TPB components.*

**H3a.**  *A fear of falling significantly affects attitudes toward AFHM.*

**H3b.**  *A fear of falling significantly affects subjective norms toward AFHM.*

**H3c.**  *A fear of falling significantly affects perceived behavioral control toward AFHM.*

**H4.**  *A fear of falling indirectly affects AFHM intention mediated by TPB components.*

**H4a.**  *A fear of falling indirectly affects AFHM intention mediated by attitude toward AFHM.*

**H4b.**  *A fear of falling indirectly affects AFHM intention mediated by subjective norm toward AFHM.*

**H4c.**  *A fear of falling indirectly affects AFHM intention mediated by perceived behavioral control toward AFHM.*

## 2. Literature Review

### 2.1. Fear of Falling and Age-Friendly Home Modification

Falls account for the most significant proportion of senior deaths from injuries; in fact, deaths from falls among adults aged ≥65 years in the US increased by 31% from 2007 to 2016 [34]. The experience of falling is a direct factor in the fear of falling. According to a study by Drozdick and Edelstein, 50% of seniors with a fear of falling had experienced falls [10]. A fear of falling reduces the overall life satisfaction of older adults [35,36]. Seniors with a fear of falling hesitate to perform daily living activities [37,38,39]. A study of 4031 older adults in the Netherlands has revealed that a fear of falling is strongly related to activity avoidance [36]. Consequently, a fear of falling negatively affects independent living in a community [40]. This limitation of physical activity due to a fear of falling negatively affects seniors’ psychological well-being; they experience issues such as loss of self-efficacy, avoidance of activity, and loss of confidence [39,41].

Installing age-friendly housing design features is necessary to prevent senior falls. Age-friendly housing design features may include grab bars, nonslip flooring materials, and sufficient lighting and may exclude steps and thresholds. According to an American Occupational Therapy Association study, AFHM reduces the fear of falling and enhances physical capacities, such as balance and strength [42]. In particular, AFHM is effective for older adults experiencing difficulty in accomplishing daily living activities as a result of a fear of falling and difficulties in movement [43]. Seniors aged ≥80 years who have lived in poor housing conditions are more likely to need AFHM and show a higher intention of choosing AFHM than younger older adults [19]. However, most current houses exclude age-friendly housing design features. Thus, if an older adult experiences physical health deterioration, his or her housing environment can be a barrier and cause falls. According to the Korean Senior Survey, only 4.6% of people aged ≥65 years have age-friendly housing features in their homes [44]. Although AFHM effectively prevents senior falls, only a few older adults have implemented it. Several barriers hinder older adults from adopting AFHM, including limited information and a lack of knowledge on AFHM [21,22,45,46], budget [47], tenure [48], and an insufficient home-modification market [49]. This scarcity of AFHM leads to falls in houses that threaten the health and well-being of older adults [18].

### 2.2. Theory of Planned Behavior

Ajzen’s (1991) TPB explains the factors related to an individual’s motivation for particular behavioral intentions and behaviors [27]. According to this theory, attitudes, subjective norms, and perceived behavioral control determine a particular behavioral intention. “Attitude” refers to the evaluation of a specific behavior to produce the expected results. For example, attitude toward home modification for fall prevention refers to determining whether to take a positive or negative evaluation depending on the degree of recognition of how many falls will be avoided through home modification behavior [50]. “Subjective norm” refers to the extent to which one perceives social expectations from family or friends that make one choose a specific behavior (e.g., home modification). “Perceived behavioral control” refers to the degree at which one perceives difficulty or ease in performing a particular behavior (e.g., home modification) [50,51].

The TPB has been used to explain the decision-making processes of older adults. For example, Ahn et al. examined older adults’ intention toward aging in place by incorporating the TPB and using the three TPB constructs (i.e., attitude, subjective norm, and perceived behavioral control) as mediators between environmental factors and behavioral intention toward aging in place [29]. They revealed that the three TPB constructs significantly explained the behavioral intention to age in place. Huang applied the TPB to the home-replacement intention of older adults in senior housing [30]. He found that attitudes, subjective norms, and perceived behavioral control significantly affect the decision to move to senior housing. Adams et al. examined the variables that influence help-request intention to solve the mental health issues of older adults by applying the TPB and concluded that the three primary TPB constructs significantly affect help-request behavioral intention [52].

### 2.3. Protection Motivation Theory and Fear of Falling

The PMT explains that emotional states of fear affect attitude and behavior through risk assessment, such as perceived vulnerability and severity [31]. The PMT proposes a research model for explaining health-related behavioral intentions based on protecting oneself from threats or fears [53]. The PMT includes evaluating threats and coping with threats, which impact preventive or avoidance behavioral intention [54]. Threats are the degree of perceived vulnerability and severity, and coping includes the perception that certain health behaviors effectively prevent threats and the self-efficacy of health behaviors [53]. By applying the PMT, Preissner et al. revealed that a fear of falling is a significant factor affecting older adults’ physical activity [53]. Similarly, using the PMT, Taheri-Kharameh et al. found that protection motivation, coping evaluations, and fear are significant indicators of Iranian seniors’ fall-prevention behaviors [32].

### 2.4. Older Parents and Adult Children

Adult children affect the lives of older parents in various ways. A study on 1600 seniors aged ≥75 years in five European countries found that 75% received emotional support, transportation, gardening, housework, personal care, and financial support from their adult children [55]. The relationship between older adults and their adult children is mainly focused on the older parents’ health [56]. Older adults with age-induced disabilities receive the most care from family members while living in the same community [57]. Their lifestyles are affected by the support they receive from their adult children [58]. Previous studies have asserted that the care and roles performed by family members, especially adult children, are directly related to older adults’ physical and psychological well-being, as well as their life satisfaction [56,59,60,61]. Moreover, the opinions of adult children significantly influence older parents’ purchase and/or use of specific products [24,26,62].

## 3. Materials and Methods

### 3.1. Participants and Sampling

The target population comprised older parents and adult children living in Busan, South Korea. The older parents were individuals aged ≥75 years with more than one child who lived in their own homes instead of an institutional setting. The adult children were individuals aged 45–64 years who were not living with their parents and had at least one living parent or parent-in-law. A self-administered questionnaire was developed for a face-to-face survey after the institutional review board’s approval (PNU IRB/2022_26_HR). A survey company collected the qualified sample in March 2022. The survey company collected data through a street survey in three districts with the highest aging population in Busan, Korea, until the target number of participants was reached. A total of 600 questionnaires (300 completed by older parents and 300 completed by adult children) were analyzed.

### 3.2. Measures

Table 1 presents the primary constructs: fear of falling, the three TPB components (i.e., attitude, subjective norm, and perceived behavioral control toward AFHM), and AFHM intention. Questions for the fear of falling asked about the degree of fear of falling felt by older parents and the adult children’s concerns about their older parents’ falls. The two groups of participants were asked about the fear of falling using one question based on research by Oh et al. (2015) [40] and Thhero-Kharameh et al. (2020) [32]. Questions for attitude consisted of three questions about positive or negative attitudes toward AFHM. The questions were modified from existing studies by Ajzen (2001) [27], Lee et al. (2013) [63], and Conner et al. (2002) [64]. Questions for subjective norm asked about significant others’ perceptions of AFHM and included three questions developed based on three studies [27,63,64]. Questions for perceived behavioral control constituted three questions referring to individual perceptions of the ease or difficulty in performing AFHM, which were developed by referring to a study by Lee et al. (2013) [63]. Lastly, behavioral intention included one question asking about one’s intention to adopt AFHM if necessary, which was modified from Wiles et al. (2012) [65]. Before starting the survey, the meaning and examples of AFHM were provided using images. All questions were measured using a seven-point Likert scale.

### 3.3. Data Analysis 

Descriptive statistics were used to determine the means and standard deviations of all variables. An independent *t*-test was employed to compare the variables between the older-parent and adult-children groups. A path model analysis was used to identify the relationships among a fear of falling, the three TPB components (i.e., attitude, subjective norm, perceived behavioral control toward AFHM), and AFHM intention for the two groups. Reliability for the three TPB components was tested using Cronbach’s alpha. In addition, the mean score of each TPB component was calculated for the path analysis. For the path model fit evaluation, multiple goodness-of-fit indices were employed: the goodness-of-fit index (GFI), the adjusted goodness-of-fit index (AGFI), the comparative fit index (CFI), and the standardized root mean square residual (SRMR). GFI, AGFI, and CFI scores of ≥0.95, collectively, and SRMR scores of <0.09 were adequate cut-off values for good fits. The Statistical Package for Social Sciences and the Analysis of Moment Structures were used for data analyses. A significance level of *p* < 0.05 was selected.

## 4. Results 

### 4.1. Descriptive Analysis of Older Parents

The mean age of older parents was 80.04 years (SD = 4.04). Overall, 37.7% were male, and 62.3% were female. Regarding their marital status, 61.0% were married, 36.7% were widowed, and 2.3% were divorced or separated. About 18% of the older parents had no formal education; among the others, the highest levels of schooling attained were elementary school (40.3%), middle school (29%), or high school or postsecondary education (12.6%). They lived in single-person (29.4%), two-person (59%), or three-or-more-person (11.6%) households. Their monthly incomes were as follows: less than USD 379 (2%), USD 380–769 (35.3%), USD 770–1499 (44.3%), USD 1500–2299 (13%), or more than USD 2300 (5.5%). 

Regarding housing characteristics, 36.3% lived in high-rise multifamily housing, 35.4% lived in low-rise apartments, and 28.3% lived in single-family homes. The majority of the respondents were homeowners (85.6%), and the rest were renters (14.4%). The mean number of years since the construction of their housing was 28.65 (SD = 7.18) years, and older parents had lived in their current homes for 17.73 (SD = 7.91) years on average.

### 4.2. Descriptive Analysis of Adult Children

Among adult children, the average age was 53.52 years (SD = 4.75); 37% were male, and 63% were female. The vast majority were married (96.7%), and a few were divorced or separated (3%). The highest levels of schooling attained were middle school or lower (2%), high school (51%), or college (47%). Their households comprised two people (40.7%), three people (50.3%), four people (8%), or other numbers of people (1%). Their monthly incomes were as follows: less than USD 2999 (15.3%), USD 3000–3799 (22.7%), USD 3800–4599 (36.3%), USD 4600–5399 (20.7%), or more than USD 5400 (5%). 

In terms of housing characteristics, 66.7% lived in high-rise multifamily housing, 17.3% lived in low-rise apartments, and 16% lived in single-family homes. Most respondents were homeowners (88%), and some were renters (12%).

### 4.3. Comparison of the Variables between Older Parents and Adult Children

Table 2 shows significant differences between older parents and adult children regarding their mean values of for a fear of falling, attitude, subjective norm, perceived behavioral control, and behavioral intention. Adult children were likelier than their parents to have concerns about their parents’ falls (*t* = −8.75, *p* < 0.001). Both older parents and adult children showed higher than six points out of seven regarding attitude toward AFHM, with no statistically significant difference. In addition, no statistically significant difference was observed between the two groups in subjective norms toward AFHM. Regarding perceived behavioral control toward AFHM, both groups showed less than five points out of seven; adult children showed statistically significantly higher points compared with older parents (*t* = −3.45, *p* < 0.01). Regarding AFHM intention, older parents showed a median value lower than four points, while adult children showed a statistically significantly higher median value (*t* = −16.464, *p* < 0.001). 

### 4.4. Relationships among Fear of Falling, Attitude, Subjective Norm, Perceived Behavioral Control, and Behavioral Intention toward AFHM between Older Parents and Adult Children

This study tested the relationships among a fear of falling, the three TPB components (i.e., attitude, subjective norm, and perceived behavioral control toward AFHM), and AFHM intention for older-parent and adult-children groups using path model analyses. The GFI, AGFI, CFI, and SRMR scores were acceptable and met the recommended standards of good fit in both older parents (GFI = 0.987, AGFI = 0.801, CFI = 0.934, SRMR = 0.0529) and adult children (GFI = 0.979, AGFI = 0.689, CFI = 0.967, SRMR = 0.0600). 

**Figure 2:**
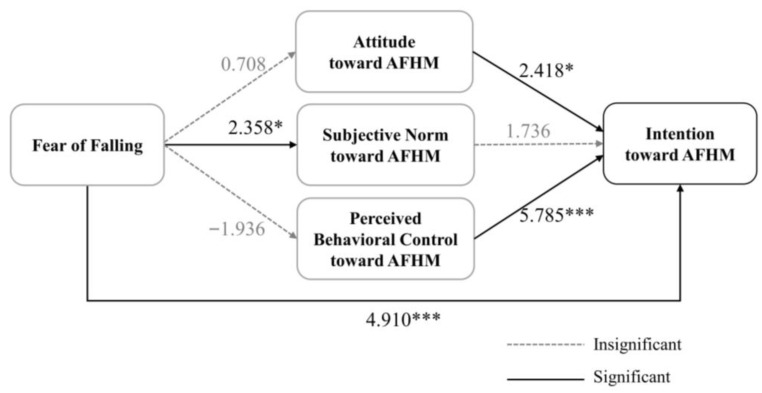
Path model results for older parents, * *p* < 0.05, *** *p* < 0.001.

For older adults, as Figure 2 shows, significant positive relationships were observed between a fear of falling and subjective norms (*r* = 2.358, *p* < 0.05) and between a fear of falling and AFHM intention (*r* = 4.910, *p* < 0.001). Attitude (*r* = 2.418, *p* < 0.05) and perceived behavioral control (*r* = 5.785, *p* < 0.001) toward AFHM were significantly positively correlated with AFHM intention. However, no statistically significant relationship was found between a fear of falling and attitude toward AFHM, between a fear of falling and perceived behavioral control toward AFHM, or between subjective norm and behavioral intention toward AFHM.

**Figure 3:**
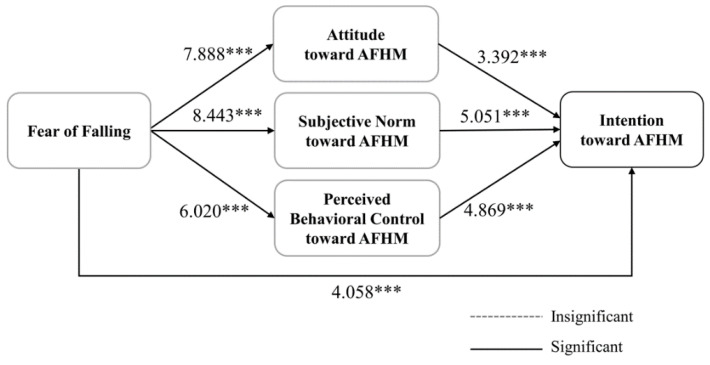
Path model results for adult children, *** *p* < 0.001.

As Figure 3 shows, all relationships in the path model were statistically significantly related in adult children. A fear of falling significantly positively affected attitude (*r* = 7.888, *p* < 0.001), subjective norm (*r* = 8.443, *p* < 0.001), perceived behavioral control toward AFHM (*r* = 6.020, *p* < 0.001), and AFMH intention (*r* = 4.910, *p* < 0.001). Moreover, attitude (*r* = 3.392, *p* < 0.001), subjective norm (*r* = 5.051, *p* < 0.001), and perceived behavioral control (*r* = 4.869, *p* < 0.001) toward AFHM were significantly positively related to AFHM intention.

## 5. Discussion

This study compared the AFHM perceptions of older parents and adult children to better understand the perceptions and decision-making processes related to AFHM intention. The noteworthy findings of this study are discussed below. 

Overall, both older parents and adult children had very positive perceptions of AFHM. While the adult children showed high intentions to choose AFHM for their parents, the older adults showed low AFHM intentions for their own home. Perceived behavioral control was the construct with the lowest mean value among TPB components for both groups, which can be related to resources such as time, budget, and AFHM information [21,22,45,46,47].

Statistically significant differences were found for the fear of falling, perceived behavioral control toward AFHM, and AFHM intention; however, no statistically significant difference was observed for attitude and subjective norm toward AFHM between older parents and adult children. Adult children were more likely to fear their parents’ falls than older parents were to fear their own. The relatively low fear of falling among the older parents in this study could suggest that they perceive a fall as a minor issue, even though 60.5% of Korean adults aged ≥65 years have experienced falls [7]. While both the older-parent and adult-children groups’ attitudes toward AFHM were positive, the adult children seemed much more intent on choosing AHFM than the older parents, which was the greatest difference among all the variables in this study. This indicates that adult children are more actively involved in AFHM than their parents. The perceived behavioral control toward AFHM of adult children was significantly higher than that of older parents. Older parents seem to perceive more barriers, such as budgets and a lack of knowledge or information about AFHM. These results support previous studies regarding the various barriers to AFHM in older adults [18,19,20,21].

This study supports using the TPB and PMT in both older-parent and adult-children groups to understand AFHM intention and behavior. The hypotheses incorporating the TPB and PMT were partially supported for the older parents’ group. A fear of falling was the second strongest predictor of AFHM intention across all three TPB components in the older parents’ group, and those who had a greater fear of falling were more likely to choose AFHM. A fear of falling also directly influenced the subjective norm, yet the subjective norm had no impact on AFHM intention. However, a fear of falling had no relation to attitude or perceived behavioral control toward AFHM. Although older parents’ attitudes toward AFHM were very positive, there was no significant impact of a fear of falling on their attitude toward AHFM, indicating that older parents may lack knowledge about the fall-prevention effects of AFHM [21,22,45,46]. Moreover, the score for a fear of falling was close to the median value and pretty low. These results may be related to a lack of information on the direct and indirect impacts of seniors’ falls on the quality of life in older adults [45,46]. Meanwhile, among the three TPB components, perceived behavioral control toward AFHM had the most substantial impact on intention toward AFHM. Older parents who felt that they had a greater ability to control AFHM had a higher AFHM intention. By contrast, those with less perceived behavioral control were less likely to choose AFHM.

All hypotheses in the proposed path model based on the TPB and PMT were supported in the adult children group. A fear of falling statistically significantly affected AFHM intention across all three TPB components and was the most strongly related to subjective norm toward AFHM. Adult children concerned about their parents’ falls were more likely to have AFHM intention, which is in line with the findings of previous studies on the fear of falling and fall-prevention behaviors using the PMT [30]. The subjective norm was the most significant indicator of AFHM intention among the three TPB components. Adult children who cared about their parents seem likelier to perceive AFHM as good behavior, leading to AFHM intention.

Unsurprisingly, older parents who fear falling and adult children concerned about their parents’ falls are more likely to choose AFHM, which supports the PMT and previous studies’ findings that older seniors living in older houses tend to need home modification [19,66]. However, the significant association between the three TPB constructs toward AFHM has crucial implications for understanding AFHM intention. Weaker attitudes and subjective norms toward AFHM that predict weaker AFHM intention may result from a lack of knowledge or information [21,22,45,46]. Lower perceived behavioral control toward AFHM that impacts lower AFHM intention would be related to a limited budget [47], human resources [49], or housing status [48]. To compensate for these weaknesses, proper support programs are needed. For example, fall-prevention and home-modification educational programs can encourage AFHM intention [45]. Supporting AFHM costs and developing AFHM educational programs or campaigns for both older parents and adult children would be helpful. 

## 6. Conclusions

This study confirmed that adult children play a critical role in fostering AFHM in an aging society. Adult children are more likely to be concerned about their parents’ falling and show positive perceptions of AFHM and high AFHM intention. This study identified two strong variables among the indirect impacts on AFHM intention. Perceived behavioral control toward AFHM had the most significant impact on AFHM intention in the older-parent group, and subjective norm was the most significant indicator of behavioral intention. Perceived behavioral control toward AFHM may be related to the personal characteristics of older parents, such as income and homeownership [18,20]. Subjective norms toward AFHM for adult children can be formed by the perception of AFHM in society. Thus, appropriate strategies for each group need to be considered to enforce AHFM. For example, policymakers can expand AFHM-supporting programs, including monetary and human-force assistance for older parents. Educational programs and public service advertisements are needed for both older adults and adult children to increase social awareness of AFHM as a public health issue. In addition, a long-term plan to expand the AFHM industry should be revitalized for older adults seeking AFHM services in the market.

This study found that the theoretical implication of incorporating the TPB and PMT to explain the behavioral process of AFHM intention is meaningful. We attempted to address the critical role of adult children in AFHM for older adults, in addition to that of older parents, by incorporating the TPB and PMT. The TPB and PMT were partially supported in the older-adult group and fully supported in the adult-children group.

## 7. Limitations and Future Studies

Although this study provides meaningful insights into understanding the perceptions and intentions of older adults and adult children with regard to AFHM, it has certain limitations and suggestions for future studies. First, this study excluded an analysis of the sociodemographic and housing characteristics of older adults and adult children, since it focused on the proposed research model. Further analysis regarding the relationship between personal characteristics and perception of AFHM would provide more specific AFHM strategies. Second, this study had limitations related to data collection. For example, this study’s older parents and adult children were not the same family members. Moreover, since the sample of this study was collected in certain areas in Busan, South Korea, it cannot be generalized to all South Korean seniors or adult children. Third, although explanations of AFHM were provided in the survey questionnaires, the range of knowledge of AFHM held by participants was not determined in this study. Depending on the extent of AFHM knowledge, older adults and adult children may have different perceptions and intentions toward AFHM. Lastly, we asked about perceived behavioral control and did not include specific elements. Further studies should identify the barriers against choosing AFHM and examine specific strategies to support the difficulties of choosing AFHM.

## Figures and Tables

**Table 1:** Instruments.

Construct	Older Parents	Adult Children	References
**Fear of Falling** ^a^	I have a fear of falling in my house.	I’m afraid my parent(s) might fall at their house(s).	Oh et al. (2015) [40] Taheri-Kharame et al. (2020) [32]
**Attitude Toward AFHM**	Overall, I think AFHM to prevent falls is … Bad (1)—Good (7) Undesirable (1)—Desirable (7) Unimportant (1)—Favorable (7)		Ajzen (2001) [27] Lee et al. (2013) [63] Conner et al. (2002) [64]
**Subjective Norm** **Toward AFHM** ^a^	Most people I know will think AFHM is a good idea.	Most people I know will think that my doing AFHM for my parent(s) is a good idea.	Ajzen (2001) [27]
	Most people who are important to me think I should choose AFHM.	Most people who are important to me think I should choose AFHM for my parent(s).	Lee et al. (2013) [63]
	People whose opinions I value would prefer that I choose AFHM.	People whose opinions I value would prefer that I choose AFHM for my parent(s).	Conner et al. (2002) [64]
**Perceived Behavioral Control** **Toward AFHM**	How confident are you that you could choose AFHM if you wanted to? ^b^	How confident are you that you could choose AFHM for your parent(s) if you wanted to? ^b^	Lee et al. (2013) [63]
	To what extent is it up to you whether you choose AFHM? ^c^	To what extent is it up to you whether you choose AFHM for your parent(s)? ^c^	
	It is my responsibility to decide whether to choose AFHM. ^a^	It is my responsibility to decide whether to choose AFHM for my parent(s). ^a^	
**Intention** **Toward AFHM** ^a^	I intend to choose AFHM if necessary.	I intend to choose AFHM for my parent(s) if necessary.	Wiles et al. (2012) [65]

Note: ^a^ = Strongly disagree (1)—strongly agree (7), ^b^ = not at all confident (1)—extremely confident (7), ^c^ = not at all (1)—great extent (7).

**Table 2:** Comparison of older parents and adult children (N = 600).

Construct	Older Parents (n = 300)	M(SD)	Adult Children (n = 300)	M(SD)	Point Estimate
Fear of Falling	I have a fear of falling in my house.	4.15 (1.32)	I am afraid my parent(s) might fall at their house(s).	5.09 (1.32)	−8.75 ***
Attitude Toward AFHM	Cronbach’s alpha = 0.72	6.14 (0.47)	Cronbach’s alpha = 0.79	6.22 (0.56)	−1.87
	Overall, I think AFHM to prevent falls is Bad (1)—Good (7)	5.97 (0.54)	Overall, I think AFHM to prevent falls is Bad (1)—Good (7)	5.97 (0.63)	
	Overall, I think AFHM to prevent falls is Undesirable (1)—Desirable (7)	6.15 (0.61)	Overall, I think AFHM to prevent falls is Undesirable (1)—Desirable (7)	6.27 (0.64)	
	Overall, I think AFHM to prevent falls is Unimportant (1)—Favorable (7)	6.31 (0.61)	Overall, I think AFHM to prevent falls is Unimportant (1)—Favorable (7)	6.43 (0.71)	
Subjective Norm Toward AFHM	Cronbach’s alpha = 0.83	5.74 (0.71)	Cronbach’s alpha = 0.87	5.85 (0.77)	−1.75
	Most people I know will think AFHM is a good idea.	5.53 (0.66)	Most people I know will think that my doing AFHM for my parent(s) is a good idea.	5.59 (0.78)	
	Most people who are important to me think I should choose AFHM.	5.79 (0.92)	Most people who are important to me think I should choose AFHM for my parent(s).	5.89 (0.85)	
	People whose opinions I value would prefer that I choose AFHM.	5.91 (0.90)	People whose opinions I value would prefer that I choose AFHM for my parent(s).	6.07 (0.93)	
Perceived Behavioral Control Toward AFHM	Cronbach’s alpha = 0.86	4.60 (0.88)	Cronbach’s alpha = 0.84	4.84 (0.74)	−3.45 **
	How confident are you that you could choose AFHM if you wanted to?	4.58 (0.88)	How confident are you that you could choose AFHM for your parent(s) if you wanted to?	5.00 (0.86)	
	To what extent is it up to you whether you choose AFHM?	4.62 (1.07)	To what extent is it up to you whether you choose AFHM for your parent(s)?	4.87 (0.80)	
	It is my responsibility to decide whether to choose AFHM.	4.62 (1.05)	It is my responsibility to decide whether to choose AFHM for my parent(s).	4.65 (0.89)	
Intention TToward AFHM	I intend to choose AFHM if necessary.	3.86 (1.14)	I intend to choose AFHM for my parent(s)if necessary.	5.22 (0.85)	−16.46 ***

M = mean, SD = standard deviation, 7-point Liker scale: 1 (strongly disagree) to 7 (strongly agree), ** *p* < 0.01, *** *p* < 0.001.

## Data Availability

Not available.
